# Tuning the morphology of sulfur–few layer graphene composites *via* liquid phase evaporation for battery application†

**DOI:** 10.1039/d1na00733e

**Published:** 2022-01-13

**Authors:** Eleonora Venezia, Lorenzo Carbone, Francesco Bonaccorso, Vittorio Pellegrini

**Affiliations:** a IIT Graphene Labs, Istituto Italiano di Tecnologia Via Morego 30 16153 Genova Italy lorenzo.carbone@lithium.plus; b BeDimensional S.p.a. Via Albisola 121 16153 Genova Italy

## Abstract

A comparative study on sulfur-based composite electrodes comprising different few-layer graphene contents prepared *via* a facile evaporation method is presented here. The active material production process employed here, exploring different sulfur–few layer graphene ratios, enabled tuning and optimization of the sample morphology, as confirmed *via* a scanning electron microscopy study. The results reveal that the graphene content is a crucial parameter yielding an optimized morphology of spherical particles composed of an elemental sulfur inner core covered by the carbonaceous compound. The electrodes are characterized in lithium metal half-cells in terms of cyclic voltammetry, galvanostatic cycling tests, rate capability and electrochemical impedance spectroscopy. Moreover, the lithium-ion diffusion coefficients of each sample are obtained by the Randles–Sevcik equation in order to evaluate the reliability of the electrochemical processes. The lithium metal half-cell with the sulfur carbon composite active material exploiting a spherical particle morphology delivers a high specific capacity of 950 mA h g^−1^ after 100 cycles at C/4 with a coulombic efficiency of 98%. An optimized sample, tuned in terms of sulfur content and morphology, shows superior performance, exhibiting capacities of 1128 mA h g^−1^ and 842 mA h g^−1^ over 80 cycles at C/4 and 2C, respectively.

## Abstract 2

A comparative study on sulfur-based composite electrodes comprising different few-layer graphene contents prepared *via* a facile evaporation method is presented here.

## Graphical abstract

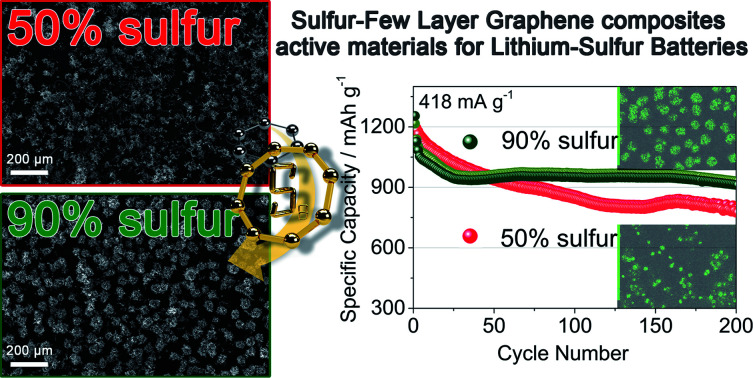

## Introduction

The recent technological progress in the energy field leading to electric vehicles, smart energy grids, and renewable energy sources has resulted in a high demand for portable and stationary energy with high reliability and low cost.^1^ Lithium-ion batteries are currently a well-known technology that has been industrially adopted in high-tech fields in a wide range of applications and established in daily usage of an increasing variety of commercial products.^2^ These systems exploit insertion material compounds, which intrinsically limit the energy density and storage capacity of the battery.^3^ Indeed, one of the most widespread Li-ion cells uses LiCoO_2_ (LCO) as the cathode material, delivering a specific capacity of 150 mA h g^−1^ and a theoretical energy density of about 550 W h kg^−1^.^4^ Recently, nickel cobalt manganese oxide cathodes (such as LiNi_0.33_Co_0.33_Mn_0.33_O_2_) with a theoretical specific capacity of 280 mA h g^−1^ and a theoretical energy density of 1036 W h kg^−1^ have emerged in addition to LCO in the Li-ion battery market. These insertion cathodes contain the cobalt element, which is derived from a rare, expensive and toxic compound.^5^ However, alternative materials, such as conversion-type materials, are required in order to achieve the energy and power densities to meet the market needs. Therefore, the lithium–sulfur battery system is intensively studied and is considered a promising choice for the next generation of energy storage devices.^6^

Lithium–sulfur technology exploits a conversion-reaction mechanism^7^ with a theoretical specific capacity of 1675 mA h g^−1^ and theoretical energy density of about 2600 W h kg^−1^.^8^ Moreover, elemental sulfur is abundant in nature, inexpensive and non-toxic, revealing this appealing system as a promising candidate for an environmental friendly energy storage solution.^9,10^ In addition to these remarkable advantages, this system suffers from considerable issues, requiring further breakthroughs in order to make the lithium–sulfur battery a practical and accessible technology. Indeed, the spontaneous reaction of lithium with sulfur evolves in the production of lithium polysulfide (Li_2_S_*x*_, *x* = 1–8) moieties.^11^ These products are soluble in common liquid organic electrolytes,^12,13^ and their dissolution may result in active material losses along cycles; this could cause capacity fading and increased cell resistance due to the insulating nature of the polysulfide species.^14,15^ Furthermore, during the charge process, the soluble species may migrate through the electrolyte and react on the lithium metal surface, leading to an electrochemical short-circuit, known as the shuttle effect.^16,17^ Moreover, elemental sulfur suffers from poor electrical conductivity due to its insulating nature, and a conductive additive is required in order to enhance the electrode conductivity.^18^ Indeed, many efforts have been devoted to the investigation and characterization of a bare elemental sulfur electrode that can fulfil market performance requirements, without positive results.^19^ Therefore, in order to overcome the abovementioned challenges, the main approaches are the investigation of new electrolyte design, the optimization and modelling of the cathode material morphology,^20^ and the use of catalysts.^21–23^ Indeed, an intense research effort has been devoted to tailor the electrode morphology by encapsulating sulfur^24,25^ into carbonaceous materials such as carbon nanotubes,^18^ graphene^20^ and porous hollow carbon spheres,^26^ acting as a matrix host to ensure good electrical conductivity within the electrode and to mitigate the dissolution of the polysulfide species through the electrolyte.^27–29^

The study herein presented reports a simple and sustainable production method of a sulfur–carbon composite active material for lithium–sulfur batteries. The production process of the active materials is based on a liquid phase evaporation method, which consists of the dispersion of few-layer graphene (FLG) flakes and elemental sulfur in an eco-friendly solvent, such as ethanol, followed by solvent evaporation. The graphene employed in this work was synthetized in powder form by a process based on wet-jet milling, as reported elsewhere.^30^

The composite production method herein disclosed allows for the tuning of the sample morphology by handling various parameters, such as the sulfur–carbon weight ratio, temperature and pressure, leading to the optimization of the active material in order to enhance its electrochemical performance. The sulfur–carbon active materials were characterized in terms of structure by X-ray diffraction measurements (XRD), morphology using scanning electron microscopy (SEM), and thermal properties *via* thermogravimetric analysis (TGA). Moreover, the active materials with different sulfur : carbon ratios were characterized in lithium metal half-cells by galvanostatic cycling tests, cyclic voltammetry, rate capability and electrochemical impedance spectroscopy. Furthermore, the lithium diffusion coefficient in each sample was calculated by the Randles–Sevcik equation.

The electrodes with sulfur : carbon ratios of 80 : 20 and 90 : 10 displaying an optimized spherical morphology exhibit specific capacities of 895 and 950 mA h g^−1^ after 100 cycles at C/4, respectively. Moreover, further optimization of the production process led to an optimized sample which delivers a specific capacity of 1128 mA h g^−1^ at C/4 and of 842 mA h g^−1^ at 2C.

## Experimental section

### Electrolyte preparation

The electrolyte solution was prepared in an argon-filled glovebox by dissolving 1 mol kg^−1^ of bis(trifluoromethane)sulfonimide lithium salt (LiTFSI) and 0.5 mol kg^−1^ of lithium nitrate (LiNO_3_) from Sigma Aldrich in 1,2-dimethoxyethane (DME) and 1,3-dioxolane (DOL) from Sigma Aldrich in a 1 : 1 weight ratio solution. Before mixing, the solvents were dried over molecular sieves for several days, while the salts were dried under vacuum at 50 °C for 24 h. The as-prepared solution was stirred overnight before assembly of the cells.

### Electrode preparation

Sulfur–few layer graphene composites were prepared *via* solvent evaporation method with ethanol as the solvent, elemental sulfur from Sigma Aldrich and few-layer graphene (indicated hereafter as FLG) obtained through the wet jet milling process of graphite powder from Sigma Aldrich, as already reported elsewhere.^30^

Elemental sulfur and FLG were mixed in powder forms in ethanol in order to obtain five samples with sulfur : FLG mass ratios of 50 : 50, 60 : 40, 70 : 30, 80 : 20 and 90 : 10, named S50FLG50, S60FLG40, S70FLG30, S80FLG20 and S90FLG10, respectively. Moreover, the characterization of the composites led to a sample with an optimized morphology, named S60OPT (60 : 40 = sulfur : FLG). The mixtures were sonicated in a sonic bath until complete sulfur dissolution. Subsequently, the solvent was slowly evaporated at 60 °C under light vacuum pressure of 400 mbar.

The preparation of the electrodes was carried out by mixing sulfur–FLG active materials with Super P carbon from Imers, multi-walled carbon nanotubes from Sigma Aldrich as conductive agents, polyvinylidene difluoride (PVdF) from Solvay as a binder in a 80 : 5 : 5 : 10 weight ratio and *N*-methylpyrrolidone (NMP) from Sigma Aldrich. The slurries were casted by doctor-blade onto a carbon cloth current collector (AvCarb) and dried overnight at 40 °C. The electrode foils were punched into 1 cm diameter disks, dried under vacuum at room temperature overnight and transferred into an argon-filled glovebox to assemble the cells. The sulfur loading of the electrodes was ∼2 mg cm^−2^.

### Material characterization

The composition of the sulfur–carbon active materials was confirmed by thermogravimetric analysis (TGA) using a Q500 thermogravimetric analyzer from TA Instruments, from room temperature to 700 °C at 5 °C min^−1^ under nitrogen flow. X-ray diffraction (XRD) measurements were performed using a Malvern PANalytical Empyrean instrument provided with a Cu Kα source in the 2*θ*/*θ* scanning mode in order to evaluate the sample structures. The morphology of the sulfur–carbon composites was investigated using a JEOL JSM-6490LA analytical scanning electron microscope (SEM) with a thermionic source (W filament).

### Cathode characterization

The electrochemical performance was tested using CR2032 coin cells. The lithium–sulfur half cells were assembled in an argon-filled glovebox with lithium metal as the anode and 2400 Celgard disks as the separator, soaked with 20 μl of DOLDME–LiTFSI–LiNO_3_ electrolyte.

Cyclic voltammetry tests were performed with a scanning rate of 0.1 mV s^−1^ in a potential range between 1.7 V and 2.8 V using a BCS Biologic instrument. The lithium ion diffusion coefficient (*D*) of the sulfur carbon composites was evaluated according to the Randles–Sevcik equation^31^ (eqn (1)) by cyclic voltammetry tests carried out at different scan rates (0.05, 0.10, 0.15, 0.20, 0.25, 0.30, 0.35, 0.40 and 0.45 mV s^−1^) in the 1.7–2.8 V voltage range using a BCS Biologic instrument as well. The lithium-ion diffusion coefficient (*D*) of each electrode was calculated by eqn (1):1
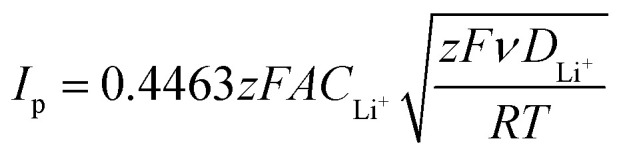
where *I*_p_ is the peak current (A), *z* is the number of electrons exchanged in the oxidation/reduction process, *F* is the Faraday constant (C mol^−1^), *A* is the active surface area of the electrode (cm^2^), *C*_Li^+^_ is the lithium-ion concentration in the active material (mol cm^−3^), *ν* is the voltage scan rate (V s^−1^), *D*_Li^+^_ is the Li^+^ diffusion coefficient (cm^2^ s^−1^), *R* is the universal gas constant (J K^−1^ mol^−1^) and *T* is the temperature (K).

An M4300 MACCOR system was employed for the galvanostatic cycling tests of the sulfur–carbon electrodes in a voltage range of 1.8–2.8 V at current rates of 1C = 1675 mA g^−1^, 2C = 3350 mA g^−1^ and C/4 = 420 mA g^−1^. The rate capability test was performed by increasing the current rate every 5 cycles from C/10 = 167.5 mA g^−1^ to 1C = 1675 mA g^−1^ through C/8 = 210 mA g^−1^, C/6 = 280 mA g^−1^, C/4 = 420 mA g^−1^, C/2 = 837 mA g^−1^, 1C = 1675 mA g^−1^ and finally back to C/10.

Electrochemical impedance spectroscopy (EIS) tests were carried out during the cyclic voltammetry tests in order to evaluate the reliability of the lithium-ion diffusion coefficient measurements in a frequency range of 1 MHz to 0.1 Hz by applying a 10 mV AC amplitude signal. In order to analyze the impedance data, the *R*_el_(*RQ*)_SEI_(*R*_ct_*Q*_dl_)*Q*_diff_ equivalent circuit was used, where *R*_el_ is the electrolyte resistance, (*RQ*)_SEI_ is the element associated with the formation of the solid electrolyte interface, *R*_ct_ is related to the charge transfer resistance, *Q*_dl_ refers to the double layer capacitance correlated to the cathode lithiation/delithiation processes and *Q*_diff_ is associated with the lithium ion diffusion within the electrode. The impedance spectra were analyzed using Boukamp software,^32^ by non-linear least squares fitting (NLLSQ), and only the results with a chi-square (*χ*^2^) value lower than 10^−4^ were accepted.

## Results and discussion

**Fig. 1:**
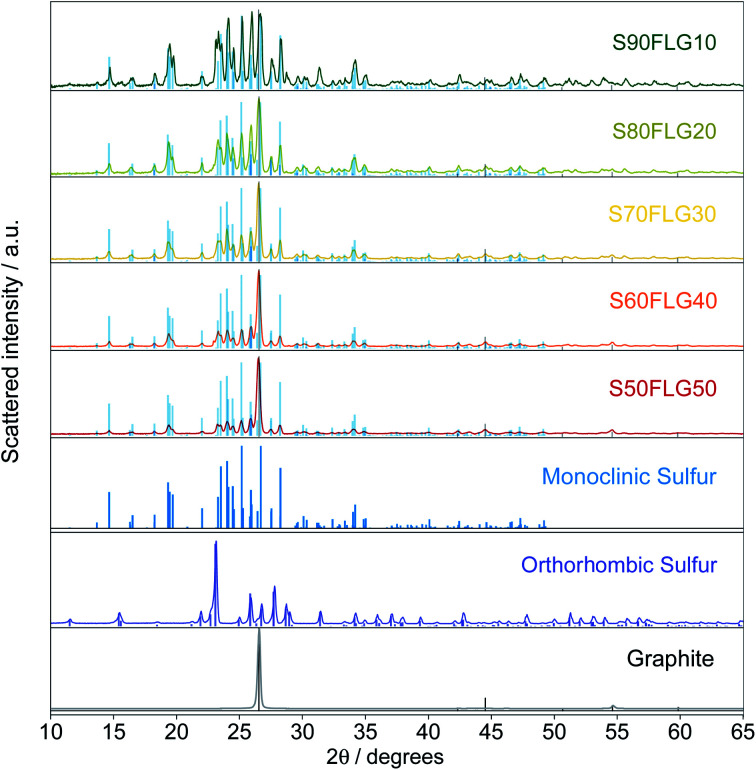
X-ray diffraction patterns of the different sulfur–few layer graphene composites, S50FLG50, S60FLG40, S70FLG30, S80FLG20, and S90FLG10, and the reference patterns of monoclinic S_8_ (ICDD: 00-013-0141), orthorhombic sulfur (ICDD: 98-020-0453) and graphite (ICDD: 04-013-0293).

The X-ray diffraction (XRD) patterns of the sulfur–few layer graphene active materials were obtained to study the phase structures of the composites (Fig. 1). The diffraction patterns of the sulfur–carbon composites reveal the presence of the characteristic peaks attributed to monoclinic sulfur (ICDD: 00-013-0141), while some of the peaks of orthorhombic sulfur are visible (ICDD: 98-020-0453), indicating the presence of both crystalline sulfur phases. The pristine sulfur employed in the synthesis of the composites shows the orthorhombic structure, thus revealing a structural modification over the production process. Indeed, since the sulfur structure remains orthorhombic in the absence of few-layer graphene (see Fig. S1 in the ESI†), the structural change may be due to the presence of FLG, indicating the intimate connection between the FLG surface and the sulfur particles. The intensities of the sulfur peaks increase from S50FLG50 to S90FLG10, as the sulfur content increases, and the peak at 2*ϑ* = 26° attributed to graphite (ICDD: 04-013-0293) is detected in each sample, revealing the slight amount of graphite composite in the FLG powder.

**Fig. 2:**
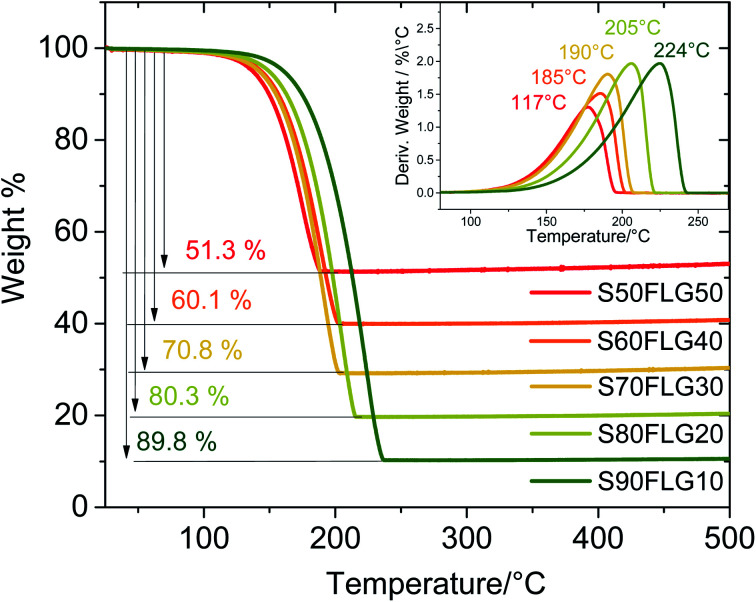
Comparison of the thermo-gravimetric analysis (TGA) of the sulfur–carbon composite samples prepared *via* liquid phase evaporation with different concentrations.

Thermo-gravimetric analysis (TGA) of the active materials was carried out in order to confirm the sulfur content of the composites. Fig. 2 reports the TGA traces of the sulfur–FLG active materials, and the corresponding differential curves are shown in the figure inset. The TGA curves of the composites show sulfur contents of 51.3 wt% in S50FLG50, 60.1 wt% in S60FLG40, 70.8 wt% in S70FLG30, 80.3 wt% in S80FLG20 and 89.8 wt% in S90FLG10. The sulfur evaporation temperature increases with decreasing FLG amount in the mixtures, ranging from 177 °C for S50FLG50 to 224 °C for S90FLG10; this reveals the possible interaction of sulfur with the carbonaceous compound.

**Fig. 3:**
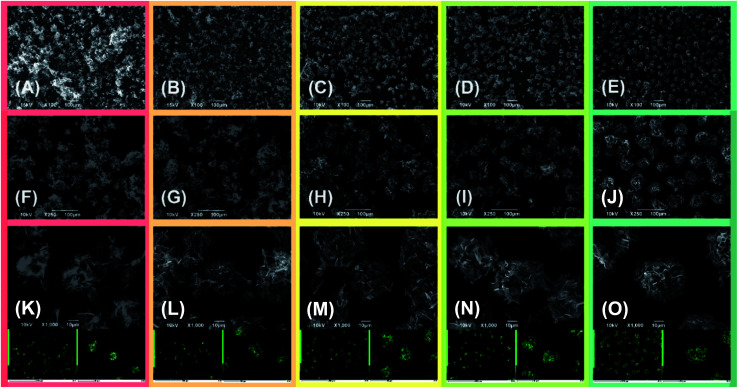
Comparison of scanning electron microscopy (SEM) images of the sulfur–FLG composites, and in the insets, the relative EDS sulfur elemental mappings of (A, F and K) S50FLG50, (B, G and L) S60FLG40, (C, H and M) S70FLG30, (D, I and N) S80FLG20, (E, j and O) S90FLG10.

**Scheme 1:**
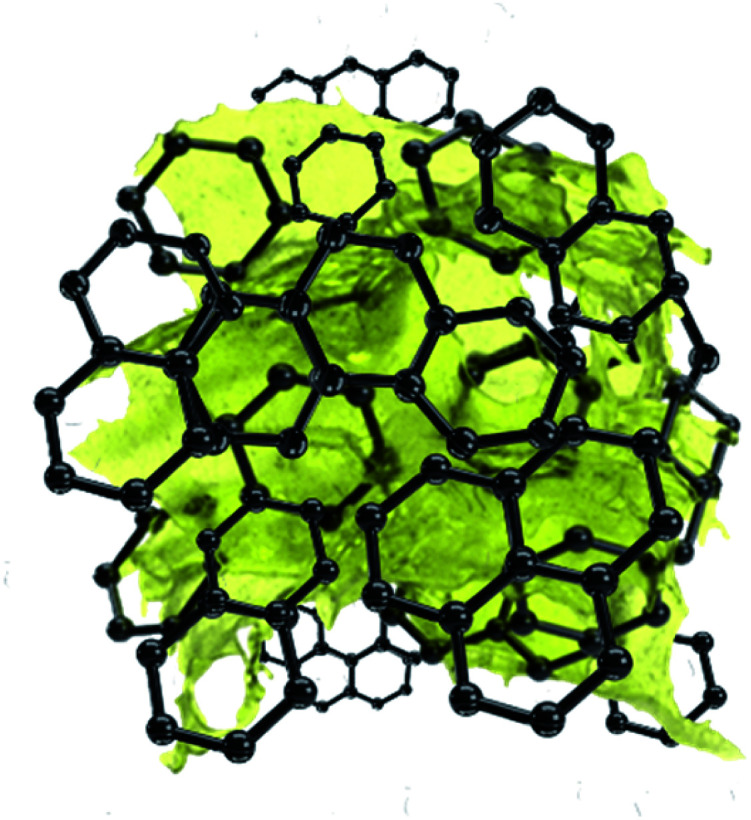
Schematic of the sulfur particle morphology, covered by few-layer graphene sheets.

The active material morphology was examined using a scanning electron microscope (SEM). The images shown in Fig. 3 for (A) S50FLG50, (B) S60FLG40, (C) S70FLG30, (D) S80FLG20 and (E) S90FLG10 were collected at lower magnification, revealing an increase of the micrometric morphology homogeneity as the sulfur content increased. In fact, the images reported in Fig. 3, (F) S50FLG50, (G) S60FLG40, (H) S70FLG30, (I) S80FLG20 and (L) S90FLG10, demonstrate the production of sulfur–few layer graphene agglomerates, which evolve into spherical sulfur–FLG particles with the increase of the sulfur : carbon ratio in the samples. Furthermore, at higher magnification (Fig. 3 (M) S50FLG50, (N) S60FLG40, (O) S70FLG30, (P) S80FLG20 and (Q) S90FLG10), the sulfur–FLG agglomerates are revealed to be composed of sub-agglomerates of about 10–20 μm, which dimension increases with the increasing of the sulfur content. However, the composite samples containing 80 wt% and 90 wt% of sulfur show a homogeneous sulfur particle dispersion with a well-defined spherical shape. The compositions of the samples were confirmed by EDS mapping, as shown in the insets to the figures, where an inner sulfur core (green shade) wrapped in the carbonaceous shell is clearly visible. This morphology could enable the retention of the polysulfide species,^33,34^ as the carbon material acts as a cage for the discharge products, and it may optimize the conduction properties within the active material,^35,36^ as shown in Scheme 1.

To test this hypothesis, an electrochemical study of the sulfur–carbon composite active materials as electrodes in energy storage devices was carried out and is reported in what follows.

**Fig. 4:**
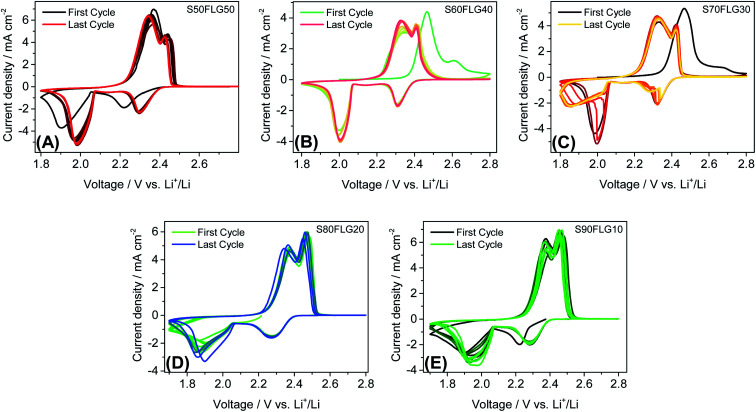
Cyclic voltammetry tests of the (A) S50FLG50, (B) S60FLG40, (C) S70FLG30, (D) S80FLG20 and (E) S90FLG10 electrodes, performed in a 2032-coin cell using DOLDME–LITFSI–LiNO_3_ as the electrolyte in the 1.7–2.8 V voltage range with a scan rate of 0.1 mV s^−1^.

The electrochemical tests were carried out using CR2032 coin cells filled with a 1 : 1 solution by weight of 1,2-dimethoxyethane (DME) and 1,3-dioxolane (DOL) containing 1 mol kg^−1^ of bis(trifluoromethane)sulfonimide lithium salt (LiTFSI) and 0.5 mol kg^−1^ of lithium nitrate (LiNO_3_). Fig. 4 displays the cyclic voltammetry tests performed on the sulfur carbon composite electrode materials in the 1.7–2.8 V voltage range at the scan rate of 0.1 mV s^−1^. The CV profiles in Fig. 4, (A) S50FLG50, (B) S60FLG40, (C) S70FLG30, (D) S80FLG20 and (E) S90FLG10, reveal the typical profile attributed to the lithium–sulfur multistep reactions. Indeed, every sulfur electrode reveals an initial reduction peak related to the reaction of elemental sulfur S_8_ to long-chain lithium polysulfides (Li_2_S_8_, Li_2_S_6_)^37^ at about 2.3 V *vs.* Li^+^/Li, followed by the formation of low-order PS species (Li_2_S_4_, Li_2_S_2_, Li_2_S) at about 1.9–2.0 V. In fact, the S50FLG50, S60FLG40 and S70FLG30 electrodes exhibit the evolution of small chain polysulfides through a narrower peak at about 2.0 V *vs.* Li^+^/Li with respect to the S80FLG20 and S90FLG10 electrodes, which react through a broader and less intense peak at 1.9 V *vs.* Li^+^/Li.^38^ The former phenomena can be ascribed to the production of a homogeneous morphology. The anodic peaks are observed at about 2.3–2.4 V *vs.* Li^+^/Li, indicating the oxidation of the short-chain polysulfides to high-order species and the further conversion to elemental sulfur.^39^ Interestingly, it is worth noting that in the samples with sulfur amounts lower than 70 wt%, the electrochemical reaction occurring at about 2.3 V exhibits a current peak higher than that of the reaction at 2.4 V, and in the samples with a higher sulfur content, the electrochemical reaction evolving at 2.4 V presents a higher current peak value; this is mainly ascribed to the complete conversion of the polysulfide moieties into elemental sulfur.^40^

**Fig. 5:**
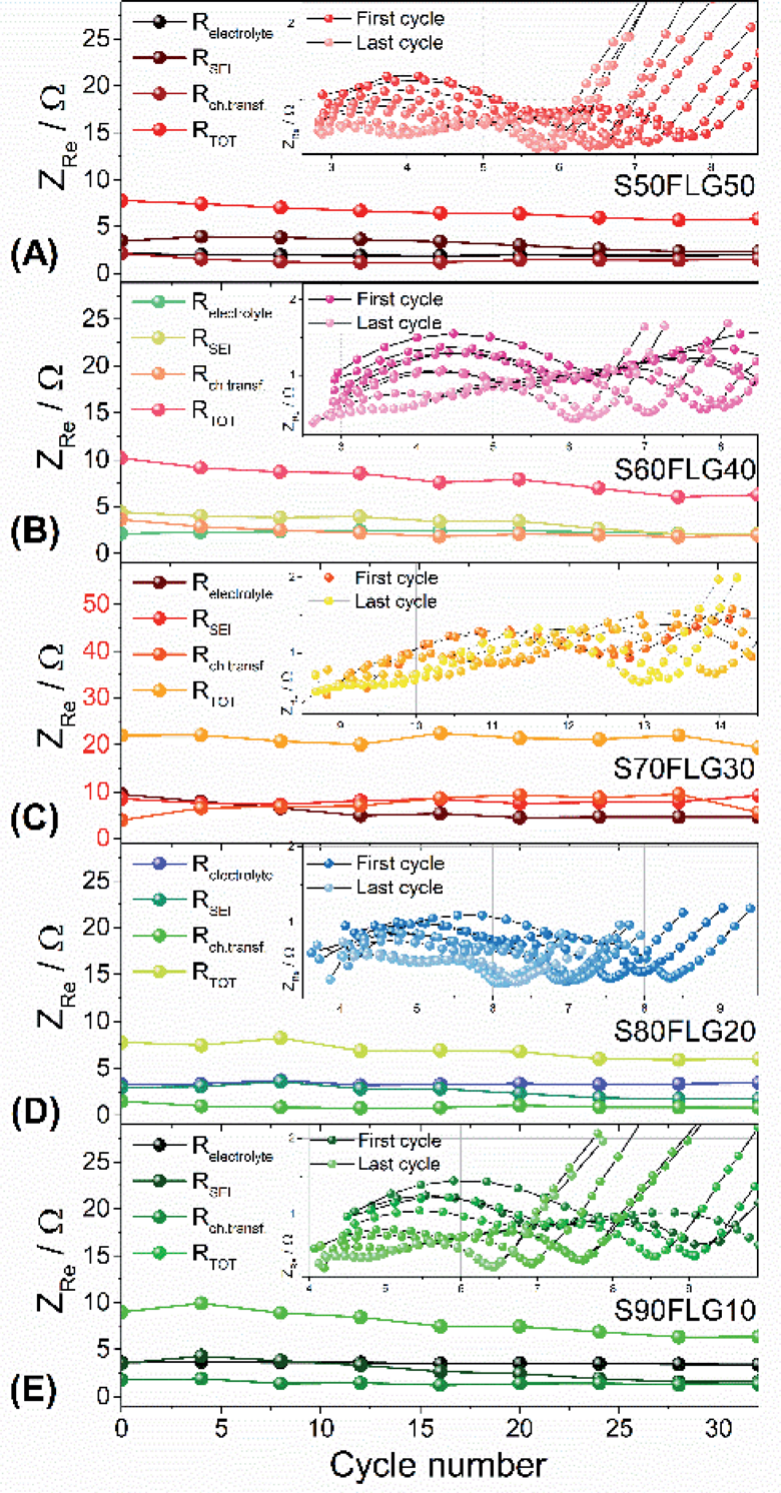
Resistance values (electrolyte, SEI, charge transfer resistance and the total value) of (A) S50FLG50, (B) S60FLG40, (C) S70FLG30, (D) S80FLG20 and (E) S90FLG10 obtained by electrochemical impedance spectroscopy (EIS), analyzed with Boukamp software. The EIS tests were carried out in a frequency range of 1 MHz to 0.1 Hz by applying a 10 mV AC amplitude signal. Insets: evolution of the EIS response during cyclic voltammetry tests performed at increasing scan rates from 0.05 mV s^−1^ to 0.45 mV s^−1^ by 0.05 mV s^−1^ in the 1.7–2.8 V voltage range.

The resistance values obtained from the electrochemical impedance spectroscopy (EIS) measurements are plotted in Fig. 5 for (A) S50FLG50, (B) S60FLG40, (C) S70FLG30, (D) S80FLG20 and (E) S90FLG10. The insets to Fig. 5 report the corresponding Nyquist plots acquired during the cyclic voltammetry tests at every scan rate step from 0.05 mV s^−1^ to 0.45 mV s^−1^ in the 1.7–2.8 V voltage range (Fig. S2 of the ESI†). Two depressed and partially overlapped semicircles in the high-medium frequency range and a straight line in the low frequency range are observed along the Nyquist plots of the impedance spectroscopy tests of all sulfur–carbon electrodes. Indeed, the semicircles are correlated with the formation of the SEI ((*RQ*)_SEI_) layer and with the charge transfer processes (*R*_ct_*Q*_dl_), while the straight line corresponds to the diffusion of Li^+^ ions within the electrode material (*Q*_diff_). The resistance evolution over the cyclic voltammetry test shows similar behavior among the samples. However, the S70FLG30 test exhibits a higher total resistance value with respect to the other samples, although multiple tests were carried out. The electrolyte resistance trend shows a stable profile over the whole test, revealing an increase of the resistance values from 2.5 Ω of the S50FLG50 sample to 4 Ω of the S90FLG10 compound. Moreover, the resistance associated with the formation of the solid electrolyte interface shows a slight increase along the first cycles due to the production of the SEI, followed by a minor decrease related to its dissolution and a subsequent stabilization process attained at about 2.5 Ω. The charge transfer resistance stands at around 1.5 Ω along the entire measure for all the samples tested except for the sample containing 70 wt% of sulfur, which shows a higher *R*_SEI_ of up to 7 Ω while the charge transfer resistance increases over cycling from 4 to 8 Ω. The aforementioned trend suggests the possible comparison between the electrode materials for further analyses, reflecting a stable and homogeneous trend among each other. Therefore, the lithium diffusion coefficients were calculated according to the Randles–Sevcik equation (eqn (1)), which correlates the peak current *I*_p_ with the square root of the scan rate, and it was determined at different states of charge, *i.e.* at 2.35 V and 2.4 V along the charge processes and at 1.9 V and 2.3 V during the discharge processes (linear fit in Fig. S3 of the ESI†). In order to calculate the diffusion coefficients, two electrons for each reduction/oxidation peak were taken into account, while the lithium ion concentration was evaluated while considering the thickness of each electrode (Fig. S4 of the ESI†).

The diffusion coefficients related to the charge and discharge processes of each electrode are summarized in Table 1 (and reported in Fig. S5(a) and (b) of the ESI†).

**Table 1:** Lithium diffusion coefficients of the electrodes

Sample	Lithium diffusion coefficient, *D*_Li^+^_/cm^2^ s^−1^			
	Charge		Discharge	
	2.35 V	2.4 V	1.9 V	2.3 V
S50FLG50	7.3 × 10^−15^	8.8 × 10^−15^	3.1 × 10^−15^	1.2 × 10^−15^
S60FLG40	2.9 × 10^−15^	2.9 × 10^−15^	1.7 × 10^−15^	5.0 × 10^−16^
S70FLG30	2.0 × 10^−15^	2.0 × 10^−15^	1.3 × 10^−15^	4.0 × 10^−16^
S80FLG20	5.2 × 10^−15^	5.1 × 10^−15^	2.4 × 10^−16^	1.1 × 10^−16^
S90FLG10	4.0 × 10^−15^	3.7 × 10^−15^	1.4 × 10^−16^	5.3 × 10^−17^

The lithium diffusion coefficient values increase with the increase of the graphene amount in all samples. This effect can be ascribed to the highly conductive properties of the carbonaceous compound, which enhance the electron charge transfer within the electrode. Moreover, the impedance spectroscopy analysis shows a slight increase of the total resistance values with the decrease of the amount of graphene in the electrode from 5.82 Ω to 6.32 Ω for S50FLG50 and S90FLG10, respectively. These values reflect the trend of the diffusion coefficients. Furthermore, the diffusion coefficients ascribed to the charge processes related to the 2.35 V and 2.4 V steps (Fig. S5(A)†) reveal higher values with respect to the diffusion coefficients of the discharge processes associated with the 1.9 V and 2.3 V steps. It is noteworthy that the trend is inverted regarding the charge reaction steps, where the samples with higher few-layer graphene amounts favor the reaction at 2.4 V, in contrast to the samples with higher sulfur contents, which promote the reaction at 2.35 V, as already observed in the cyclic voltammetry tests shown in Fig. 4.

**Fig. 6:**
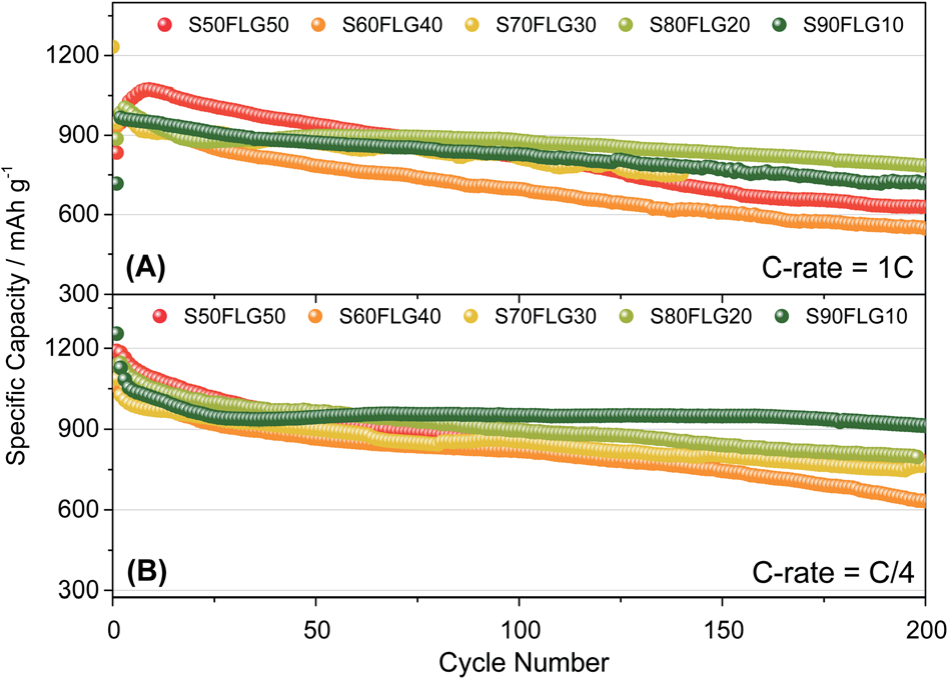
Galvanostatic cycling profiles of the sulfur–FLG electrodes performed in 2032-coin cells with DOLDME–LITFSI–LiNO_3_ electrolyte carried out at (A) 1C = 1675 mA g^−1^ and (B) C/4 = 419 mA g^−1^ current rates within the voltage limits of 1.8–2.6 V. Temperature 30 °C. Sulfur loading of ∼2 mg cm^−2^.

The galvanostatic cycling performance of the sulfur–few layer graphene composites was evaluated and compared, as shown in Fig. 6 (voltage profiles in Fig. S6 of the ESI†). The cells were tested over 200 cycles at the current rates of 1C (1675 mA g^−1^) and C/4 (419 mA g^−1^), as shown in Fig. 6(A) and (B), respectively, and the specific capacity was calculated according to the sulfur content. At a constant current density of 1C (Fig. 6(A)), the samples S60FLG40, S70FLG30, S80FLG20 and S90FLG10 reveal an initial specific discharge capacity of about 1000 mA h g^−1^, while the S50FLG50 electrode shows an activation phenomenon; this is probably due to the wettability process of the electrode exhibiting a capacity increase over the first 25 cycles, followed by a rapid decrease over 200 cycles (retention capacity at last cycle *R*_c_ = 59%). The S60FLG40 and S70FLG30 electrodes showed rapid capacity fading with respect to the samples with a higher sulfur/FLG ratio, with capacity retentions of 60% and 75% at the last cycle, respectively. The S80FLG20 and S90FLG10 electrodes show similar behavior to the former electrodes upon cycling, maintaining discharge capacities of 786 mA h g^−1^ and 720 mA h g^−1^ and thus revealing capacity retentions of 80% and 75%, respectively. At the lower current rate of C/4 (Fig. 6(B)), the samples exhibit a slight decrease of the specific capacity along the first 25 cycles, followed by a stable trend. The S50FLG50, S70FLG30 and S80FLG20 cells show initial discharge capacities of 1181, 1086 and 1137 mA h g^−1^, respectively, with a stable decreasing trend after the initial activation steps, achieving a specific capacity of about 780 mA h g^−1^. The S60FLG40 cell revealed a lower capacity retention even at a lower current rate, with an initial capacity of 1024 mA h g^−1^ and a final capacity of 631 mA h g^−1^. The sample with the highest sulfur content, S90FLG10, presents a high initial specific capacity of 1254 mA h g^−1^, which slightly decreases and finally stabilizes at about 950 mA h g^−1^ for 200 cycles (*R*_c_ = 76%). The stable behavior of the former electrode can be ascribed to the previously defined morphology of the sample (see Fig. S7 of the ESI† for SEM, EDX and XRD analyses of S80FLG20 and S90FLG10 after cycling), in which the graphene flakes cover the sulfur particles, allowing possible retention of the polysulfide species and enhancing the conduction properties along the electrode. Therefore, an optimized configuration of the sulfur graphene active material was performed in order to enhance the cell performance, combining the morphological effect and the conductive agent properties.

**Fig. 7:**
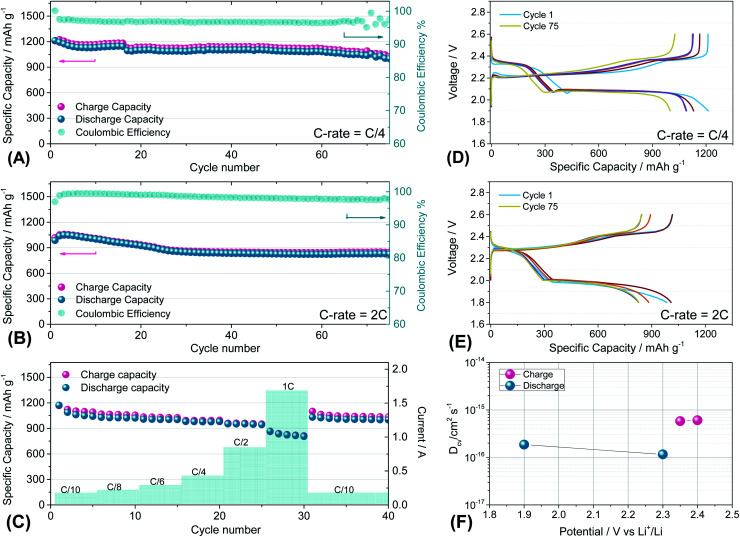
(A and B) Galvanostatic cycling profiles and (D and E) voltage profiles of the S60OPT electrode performed in 2032-coin cells with DOLDME–LiTFSI–LiNO_3_ electrolyte carried out at (A and D) C/4 = 419 mA g^−1^ and (B and E) 2C = 3350 mA g^−1^ within the voltage limits of 1.9–2.6 V and 1.8–2.6 V, respectively. (C) Rate capability test of S60OPT performed at different current rates: C/10 to 1C (=1675 mA g^−1^) through C/8, C/6, C/4, C/2 and 1C and finally back to C/10. Temperature 30 °C. Sulfur loading of ∼2 mg cm^−2^. (F) Lithium ion diffusion coefficients obtained by the CV tests carried out at different scan rates (see the ESI, Fig. S7a†) by using the Randles–Sevcik equation, the peak intensity (*I*_p_) and the scan rate (*ν*).

Fig. 7 reports the cycling trends at C/4 (Fig. 7(A) and (D)) and 2C (Fig. 7(B) and (E)) of the S60OPT sample in the 1.9–2.6 V and 1.8–2.6 V range, respectively. Fig. 7(A) reveals an initial capacity of 1210 mA h g^−1^, which decreases at about 1100 mA h g^−1^ after 15 cycles and remains stable over 50 cycles with an efficiency of 97% and a low polarization, as described in the corresponding voltage profile of Fig. 7(D). Indeed, the voltage profile of S60OPT at the current rate of C/4 reveals the standard sulfur electrochemical reactions with lithium, exhibiting an initial plateau at 2.3 V followed by a plateau at 2.1 V, which is responsible for delivering a major part of the specific capacity along the discharge processes, and a long plateau at about 2.2 V during the charge processes. At the 2C-rate (Fig. 7(B) and (E)), the cell exhibits an initial specific capacity of 1050 mA h g^−1^, with a slight decrease along the first 25 cycles at about 840 mA h g^−1^. Moreover, the specific capacity remains constant upon cycling, with a coulombic efficiency approaching 100%. However, the cell shows high cell polarization, as reported in Fig. 7(E), revealing a hysteresis of about 0.3 V between the charge and discharge main voltage plateau profiles. The rate capability test reported in Fig. 7(C) shows reversible capacities of 1167, 1124, 1008, 980, 958 and 862 mA h g^−1^ at C/10, C/8, C/6, C/4, C/2 and 1C (=1675 mA g^−1^), respectively, demonstrating good capacity recovery back to C/10 delivering about 1150 mA h g^−1^ (see Fig. S8 of the ESI† for the cumulative irreversible capacity calculations). The cyclic voltammetry and impedance measurements for S60OPT are reported in Fig. S8 of the ESI,† reflecting the conventional lithium sulfur electrochemical reactions and resistance trend of the aforementioned electrode samples. Lithium diffusion coefficients were calculated for the sample S60OPT, as mentioned before (see the ESI† for the linear fit and electrode thickness, Fig. S9(a), S10 and S11†), and they are shown in Fig. 7(F). In the charged state, the lithium diffusion coefficient values are 5.8 × 10^−16^ and 6.0 × 10^−16^ cm^2^ s^−1^ at 2.35 and 2.4 V, respectively, while those in the discharged state are 1.8 × 10^−16^ cm^2^ s^−1^ at 1.9 V and 1.1 × 10^−16^ cm^2^ s^−1^ at 2.3 V. These values lie between the lithium diffusion coefficients obtained for S80FLG20 and S90FLG10, reflecting the effectiveness of the production processes, with a slight increase due to the addition of few-layer graphene as a conductive agent.

## Conclusions

Sulfur–carbon composite materials containing few-layer graphene were synthesized with different morphologies using a simple liquid phase evaporation method. The obtained results proved the potentiality of these electrodes as lithium–sulfur cell cathodes. The study showed how the different sample morphologies obtained by tuning the amount of graphene in the composite directly influenced the electrochemical performance. Indeed, among the various sulfur–carbon composites, the samples containing higher amounts of sulfur and with a well-defined spherical particle morphology (S80FLG20, S90FLG10 and S60OPT) demonstrated the most encouraging results. The electrode containing 90 wt% of sulfur (S90FLG10) delivered a specific capacity of 950 mA h g^−1^ at C/4, demonstrating stable behavior upon cycling and thus revealing its spherical morphology an efficient way to trap the polysulfide species. Although S50FLG50 showed the highest lithium diffusion coefficient, the sample presented high capacity fading, probably due to its inhomogeneous and undefined morphology. Therefore, an optimized electrode based on the morphology and few-layer graphene content showed superior performance, delivering a specific capacity of 1128 mA h g^−1^ at C/4 and of 842 mA h g^−1^ at 2C. Although the increase of the sulfur loading remains a pivotal aspect in the step forward to the commercialization of lithium–sulfur batteries, this promising system reveals that morphology optimization is a fundamental parameter in order to increase the electrochemical performance of cells.

## Conflicts of interest

There are no conflicts to declare.

## Supplementary Material

NA-004-D1NA00733E-s001
