# Correction: Tuning the morphology of sulfur–few layer graphene composites *via* liquid phase evaporation for battery application

**DOI:** 10.1039/d3na90037a

**Published:** 2023-04-14

**Authors:** Eleonora Venezia, Lorenzo Carbone, Francesco Bonaccorso, Vittorio Pellegrini

**Affiliations:** a IIT Graphene Labs, Istituto Italiano di Tecnologia Via Morego 30 Genova Italy lorenzo.carbone@lithium.plus; b BeDimensional S.p.a. Via Albisola 121 16153 Genova Italy

## Abstract

Correction for ‘Tuning the morphology of sulfur–few layer graphene composites *via* liquid phase evaporation for battery application’ by Eleonora Venezia *et al.*, *Nanoscale Adv.*, 2022, **4**, 1136–1144, https://doi.org/10.1039/D1NA00733E.

The authors regret that the following funding information was omitted in the original manuscript:

“This project has received funding from the European Union’s Horizon 2020 research and innovation programme under grant agreement GrapheneCore3 – 881603.”

The Royal Society of Chemistry apologises for these errors and any consequent inconvenience to authors and readers.

## Supplementary Material

