# Supplementary material for: Tuning the morphology of sulfur–few layer graphene composites via liquid phase evaporation for battery application
Source: Nanoscale Adv. 2022 Jan 13;4(4):1136–44. doi: 10.1039/d1na00733e (PMC9418723; doi:10.1039/d1na00733e)
Supplement: NA-004-D1NA00733E-s001 [file NA-004-D1NA00733E-s001.pdf]

Electronic Supplementary Material (ESI) for Nanoscale Advances.
This journal is © The Royal Society of Chemistry 2022

# Tuning the Morphology of Sulfur-Few Layers Graphene Composites Via Liquid Phase Evaporation for Battery Application

Eleonora Venezia, [a] Lorenzo Carbone [a *], Francesco Bonaccorso [b] and Vittorio Pellegrini [b]

[a] IIT Graphene Labs, Istituto Italiano di Tecnologia, Via Morego 30, 16153, Genova, Italy

[b] BeDimensional S.p.a. Via Albisola 121, 16153, Genova, Italy

*Corresponding Authors: lorenzo.carbone@lithium.plus

**Keywords**

Lithium Batteries, Sustainable Energy, Sulfur-Graphene,

## Supplementary Information

Figure S1 shows the x-ray diffraction patterns of the sulfur employed in the composites preparation before and after the evaporation process. The pristine sulfur maintained its orthorhombic structure after being processed. Therefore, the sulfur structural change detected in the composite materials, in which also the sulfur monoclinic phase is present, could be ascribe to the presence of few-layer graphene flakes.

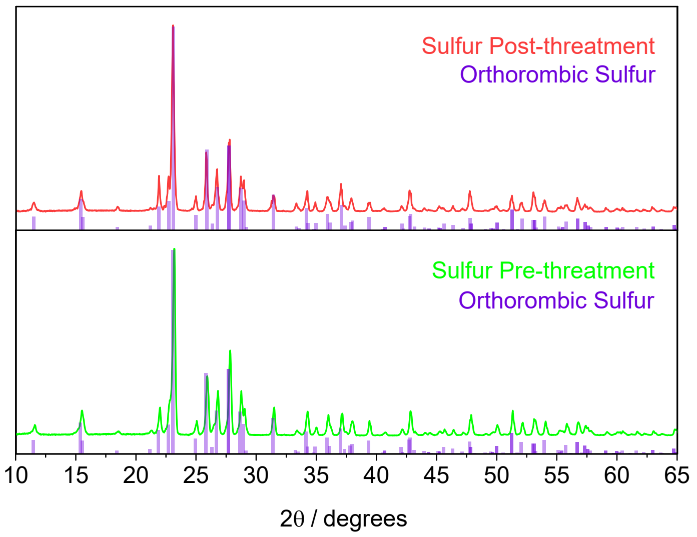


*Figure S1 - X-ray diffraction pattern of sulfur before and after the liquid phase evaporation process and reference pattern of orthorhombic sulfur (ICDD:98-020-0453).*

Figure S2 reports the cyclic voltammetry tests performed on (a) S50FLG50, (b) S60FLG40, (c) S70FLG30, (d) S80FLG20 and (e) S90FLG10 at different scan rates (0.05, 0.10, 0.15, 0.20, 0.25, 0.30, 0.35, 0.40 and 0.45 mV $s^{-1}$) in 1.7 – 2.8 V voltage range, in order to calculate the lithium ion diffusion coefficient (D) in each electrode, according to the Randles-Sevcik equation (see Manuscript Eq. 1). The CV profiles of each sample reveal the typical shape attributed to the multistep reactions of lithium with sulfur.

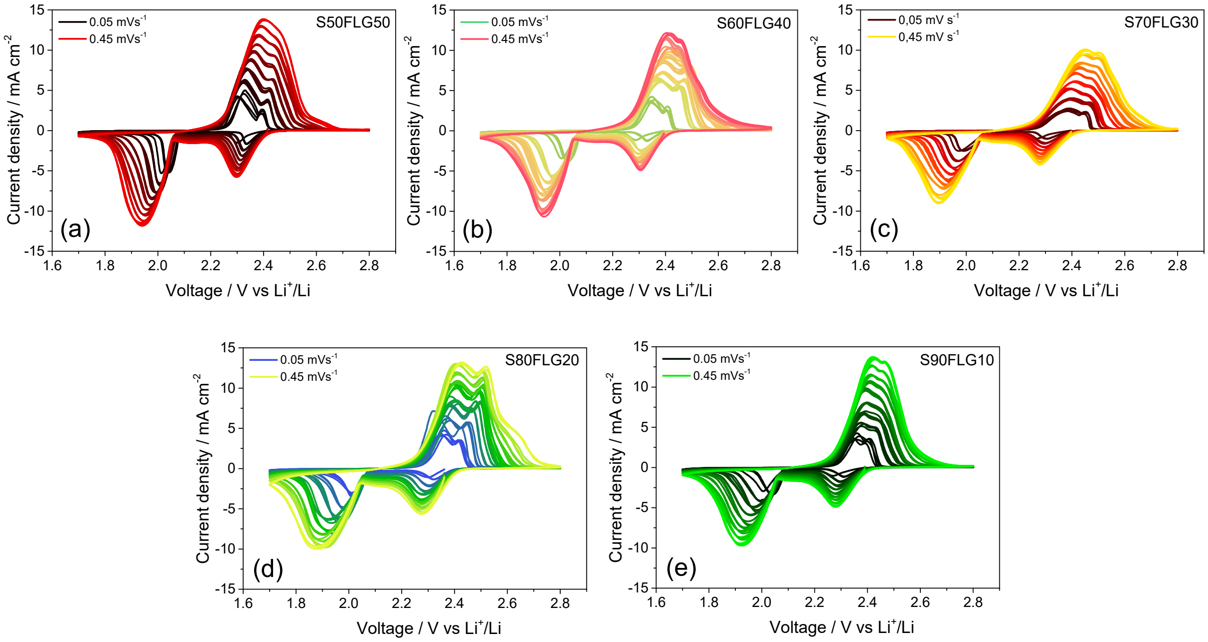


*Figure S2 - Cyclic voltammetry tests of (a) S50FLG50, (b) S60FLG40, (c) S70FLG30, (d) S80FLG20 and (e) S90FLG10 electrodes, performed in 2032-coin cell using DOLDME-LITFSI-$LiNO_3$ as electrolyte in 1.7 V – 2.8 V voltage range by increasing the scan rate, from 0.05 mV $s^{-1}$ to 0.45 mV $s^{-1}$ by 0.05 mV $s^{-1}$ every 5 cycles.*

Figure S3 represents the linear fit of charge and discharge peak current ($I_p$) vs. the square root of the scan rate ($\nu^{1/2}$) by analyzing the cyclic voltammetry profile of figure S2. For each sample (S50FLG50, S60FLG40, S70FLG30, S80FLG20, S90FLG10) are reported the fits at about 2.3 and 2.4 V vs. $Li^+$/Li during charge and the peaks at 2.3 and 1.9 V vs. $Li^+$/Li during the discharge. The slopes of the linear fits were obtained in order to calculate the lithium diffusion coefficient according to the Randles-Sevcik equation (see Manuscript Eq. 1).

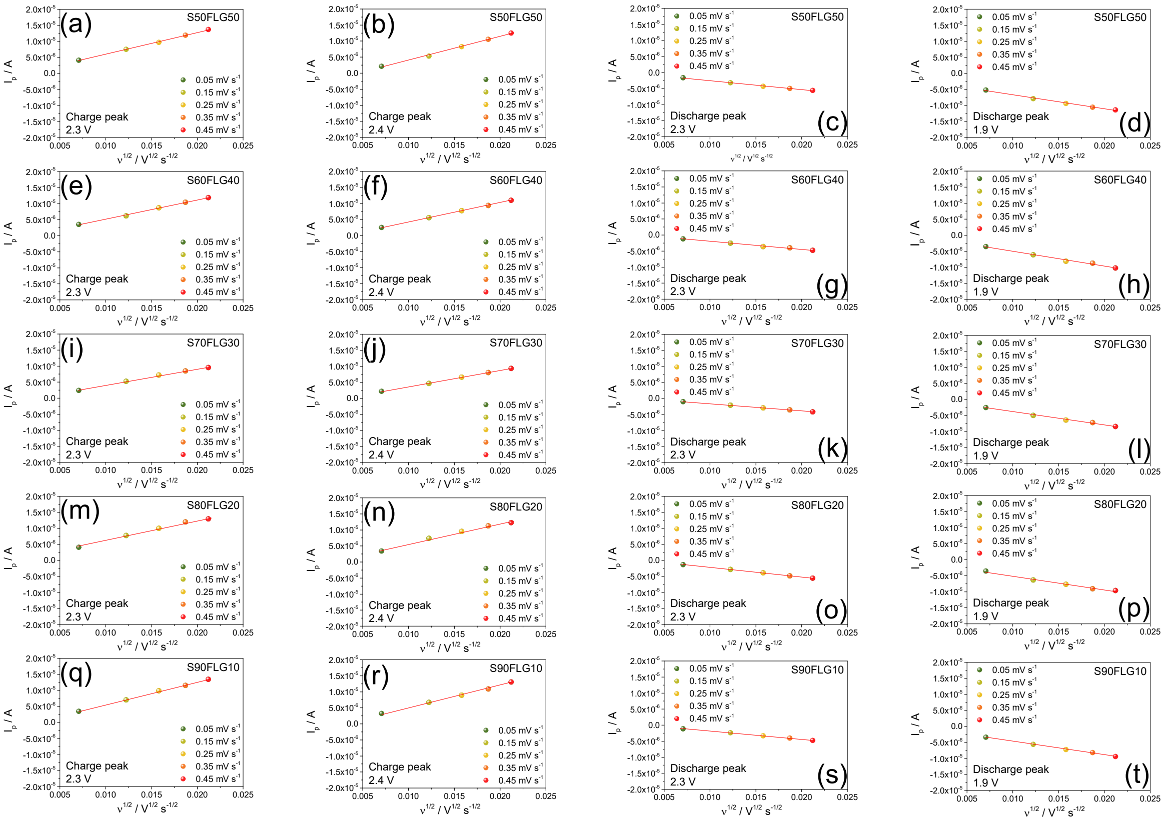


*Figure S3 - Linear fit of the peak current ($I_p$) plotted vs. square root of the scan rate ($v^{1/2}$) of the cyclic voltammetry of figure S3 of the different samples: a) S50FLG50, b) S60FLG40, c) S70FLG30, d) S80FLG20 and e) S90FLG10. From left to right, are represented the linear fit of the first charge peak at about 2.3 V involving 2 electrons, the second peak charge at 2.4 V involving 2 electrons, the first discharge peak at 2.3 V e the second peak at 1.9 V both involving 2 electrons.*

Figure S4 show the thickness of (a) S50FLG50, (b) S60FLG49, (c) S70FLG30, (d) S80FLG20 and (e) S90FLG10, measured in order to calculate the lithium-ion diffusion coefficient using Randles-Sevcik equation.

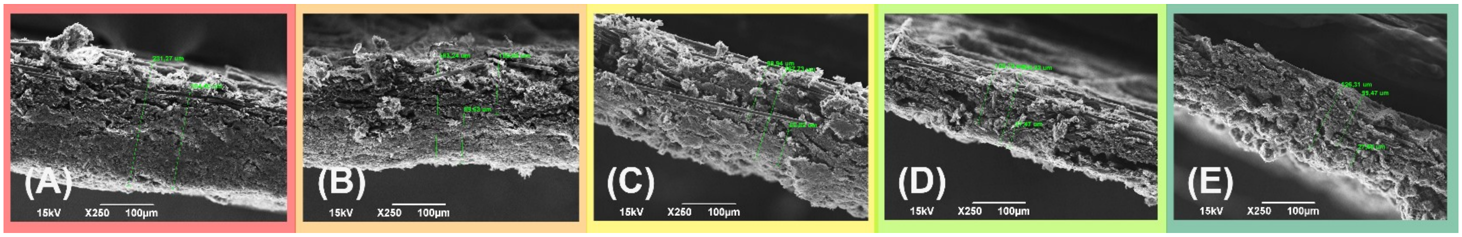


*Figure S4 - SEM images of electrode thickness of (a) S50FLG50, (b) S60FLG49, (c) S70FLG30, (d) S80FLG20 and (e) S90FLG10, respectively, measured in order to calculate the lithium-ion diffusion coefficient using Randles-Sevcik equation. See manuscript Table 1.*

Figure S5(a) ad (b) shows the lithium-ion diffusion coefficient of the five samples for charging and discharging processes, calculated accordingly to the Randles-Sevcik equation. The values are summarized in Table 1 of the Manuscript.

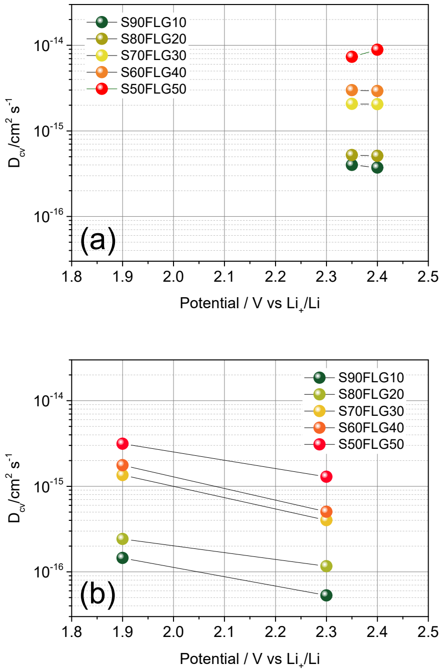


*Figure S5- (a) and (b) Lithium ion diffusion coefficient for charge and discharge processes respectively, obtained by using the Randles-Sevcik equation.*

Figure S6 shows the voltage profiles of (a),(f) S50FLG50, (b),(g) S60FLG40, (c),(h) S70FLG30, (d),(i) S80FLG20 and (e),(l) S90FLG10. From figure (a) to (e) the electrodes were tested at 1C=1675 mAhg$^{-1}$, while from (f) to (l) the measurements were carried out at a C-rate=419 mAhg$^{-1}$ (C/4). At higher C-rate (1C), the samples with lower sulfur content reveal a greater polarization with respect to the S80FLG20 and S90FLG10 electrodes. At lower C-rate, all the electrodes show a low cells polarization and an initial capacity of about 1200 mAhg$^{-1}$, which decrease in S50FLG50 while remains more stable for S90FLG10.

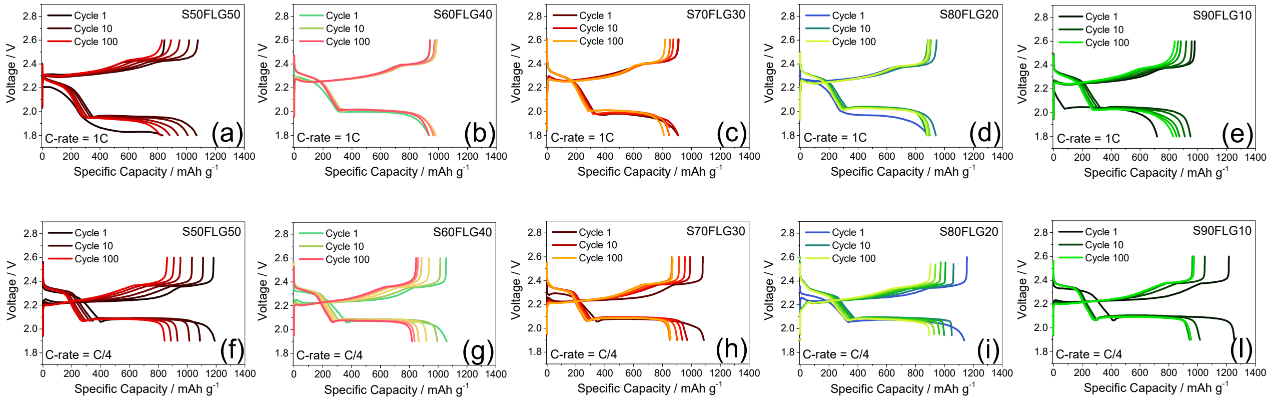


*Figure S6 - Voltage profiles of (a), (f) S50FLG50, (b), (g) S60FLG40, (c), (h) S70FLG30, (d), (i) S80FLG20 and (e), (l) S90FLG10 electrodes tested in 2032-coin cell using DOLDME-LITFSI-LiNO$_3$. (a) to (e) tests were performed at 1C=1675 mAhg$^{-1}$ in a 1.8 V – 2.6 V voltage range, (f) to (l) measurements were carried out at C/4= 419 mAhg$^{-1}$ within a voltage range of 1.9 V – 2.6V. See manuscript figure 6.*

Figure S7(a) shows the typical lithium-sulfur charge and discharge profiles, where p1 and p2 are the point at which the SEM images of the samples S90FLG10 and S80FLG20 (Figure S7(b), (c), (d) and (e)) where acquired. The XRD pattern in figure S7(f) and (g) refer to S90FLG10 and S80FLG20 samples respectively and they were acquired after cycling.

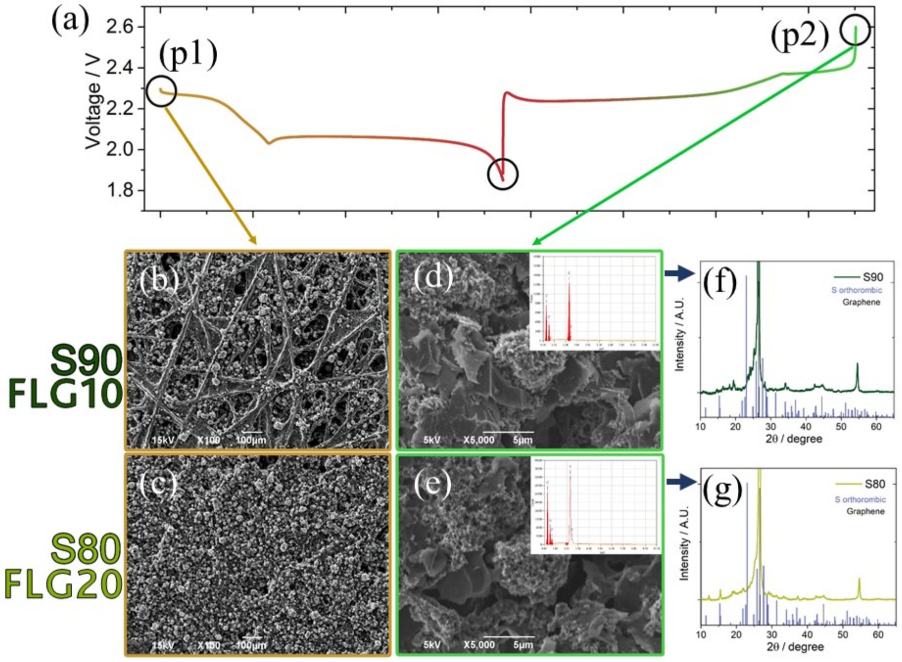


*Figure S7– (a) Charge and discharge profile of a lithium-sulfur cell, (b) and (c) SEM images of samples S90FLG10 and S80FLG20 electrodes before cycling, (d) and (e) SEM images of S90FLG10 and S80FLG20 electrodes after cycling, with EDX maps in inset, (f) and (g) X-ray diffraction pattern of S90FLG10 and S80FLG20.*

The Cumulative Irreversible Capacity (CIC) was calculate for the optimized sample (S60OPT) and the results are shown in Figure S7. At a lower current rate as C/4, after an initial increase, probably due to the consumption of electrolyte and the following SEI formation, the CIC value stabilizes around 38 mAh $g^{-1}$. At higher current rate (2C) the CIC shows a slight increase until about 20 $mAhg^{-1}$ , phenomena that may be ascribed to the  possible electrolyte consumption which reflect the activation of irreversible electrochemical processes.

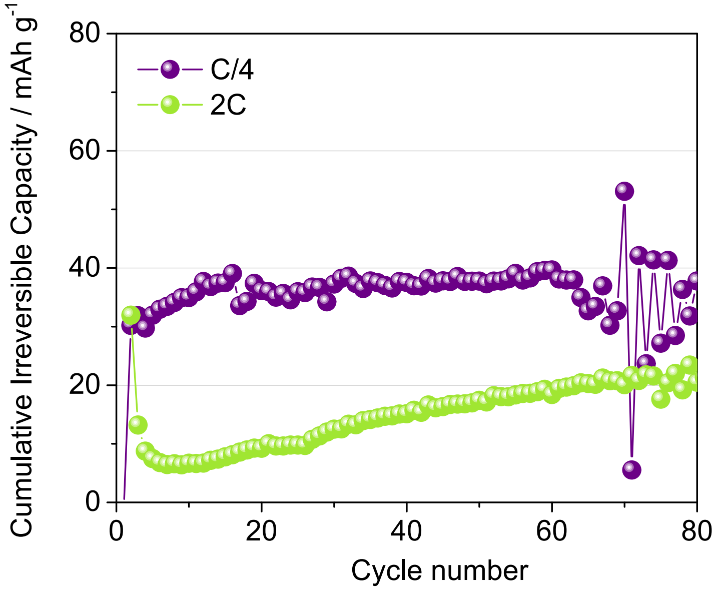


*Figure S8– Cumulative irreversible capacity at C/4 and 2C for the sample S60OPT.*

Figure S9(a) shows the cyclic voltammetry of the S60OPT sample. The test reveals the typical peaks of the multistep reactions occurring between lithium and sulfur. Figure S9(b) and (c) reports the cyclic voltammetry test performed at different scan rates (0.05, 0.10, 0.15, 0.20, 0.25, 0.30, 0.35, 0.40 and 0.45 mV $s^{-1}$) in 1.7 – 2.8 V voltage range, in order to calculate the lithium-ion diffusion coefficient (D) and the Nyquist plot of EIS tests performed throughout the voltammetry, respectively.  Nyquist plot evidences a reduction in the cell resistance, as confirmed by figure S9(d) which represent the evolution of the electrolyte, SEI and charge transfer resistances analyzed by non-linear least squares fit (NLLSQ) using Boukamp software.

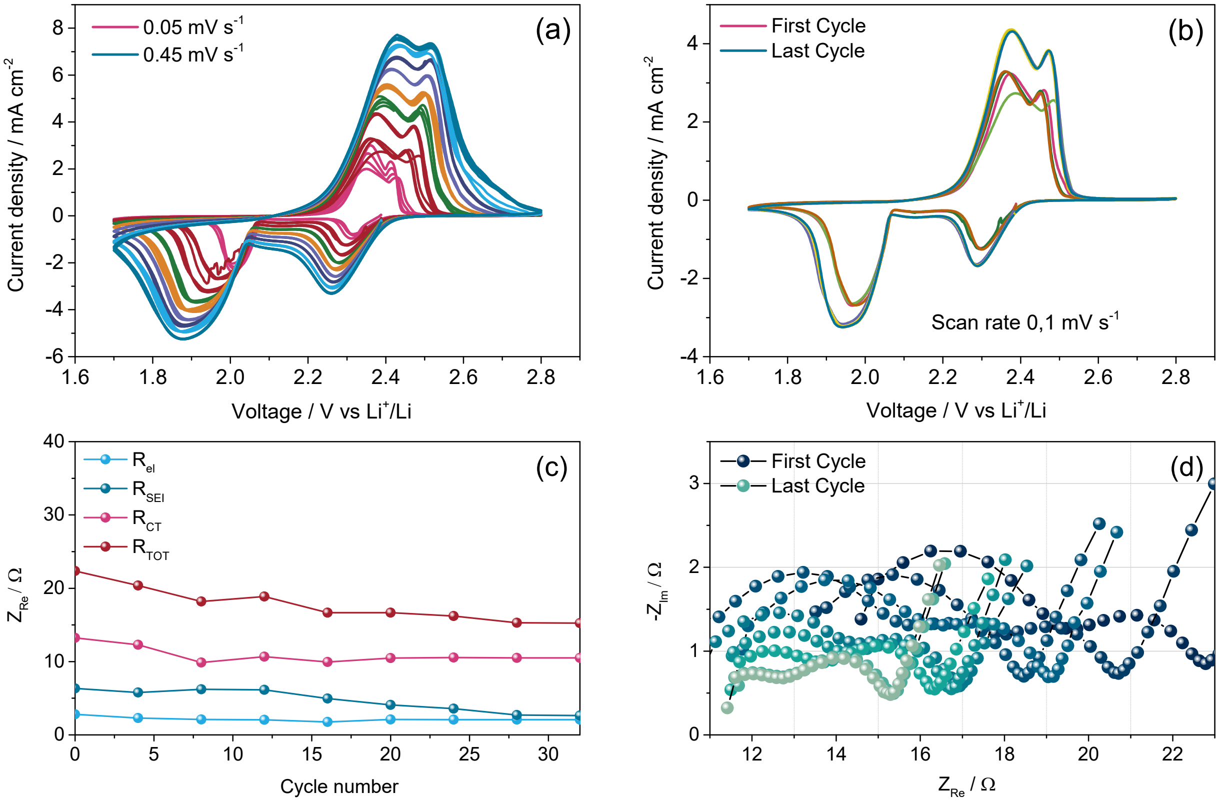


*Figure S9-(a) Cyclic voltammetry tests of S60OPT electrode, performed in 2032-coin cell using DOLDME-LITFSI-$LiNO_3$ as electrolyte in 1.7 V – 2.8 V voltage range by increasing the scan rate, from 0.05 mV $s^{-1}$ to 0.45 mV $s^{-1}$ by 0.05 mV $s^{-1}$ every 5 cycles. (b) Cyclic voltammetry test performed in the same conditions with a scan rate of 0.1 mV $s^{-1}$. (c) Resistance values (electrolyte, SEI, charge transfer resistance and the total value) of S60OPT obtained by electrochemical impedance spectroscopy (EIS), analyzed with Boukamp software. The EIS tests were carried out in a frequency range of 1 MHz – 0.1 Hz by applying a 10 mV AC amplitude signal. (d) Evolution of the EIS response at increasing scan rate, from 0.05 mV $s^{-1}$ to 0.45 mV $s^{-1}$ by 0.05 mV $s^{-1}$ every 5 cycles in 1.7 V – 2.8 V voltage range.*

Figure S10 represents the linear fit of charge and discharge peak current ($I_p$) vs. the square root of the scan rate ($\nu^{1/2}$) of the cyclic voltammetry of figure S9(b). Fig. (a),(b) report the fits at about 2.4 and 2.3V vs. $Li^+$/Li during charge, respectively and fig.(c),(d) the discharge peaks at 1.9 and 2.3V vs. $Li^+$/Li, respectively. The slopes of the linear fits were calculated in order to evaluate the lithium diffusion coefficient according to the Randles-Sevcik equation (see Manuscript Eq. 1).

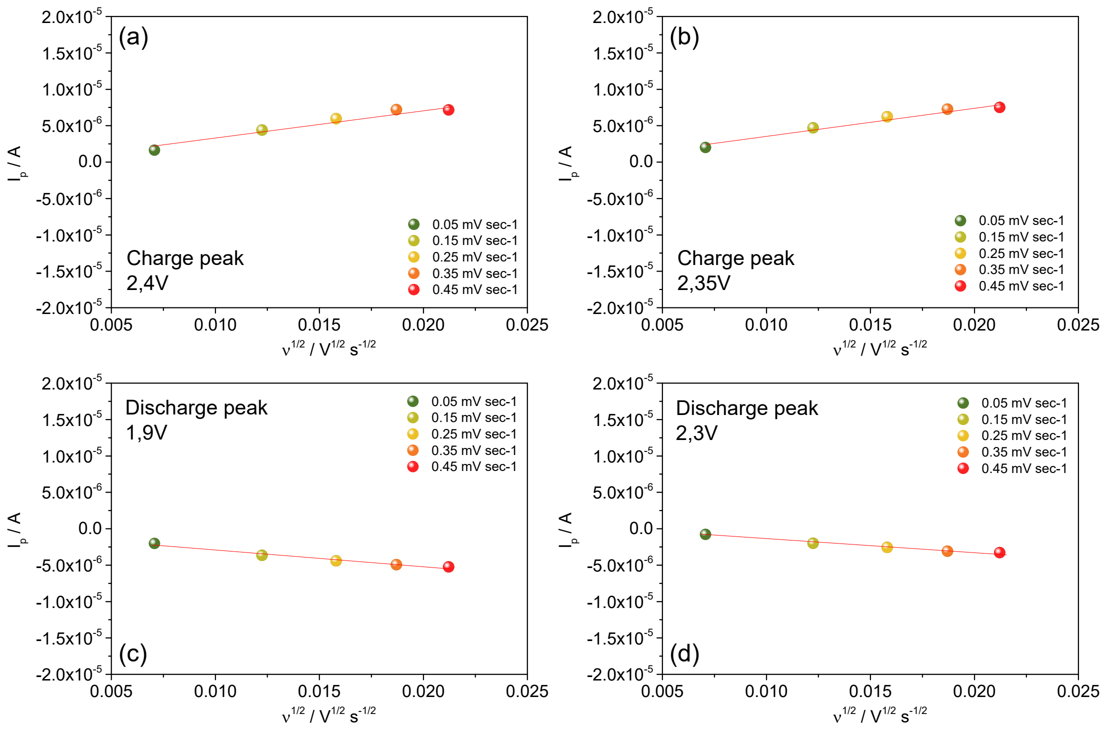


*Figure S10- Linear fit of the peak current ($I_p$) plotted vs. square root of the scan rate ($\nu^{1/2}$) of the cyclic voltammetry of figure S5 of the sample S60OPT. From left to right, are represented the linear fit of the first charge peak at about 2.3 V involving 2 electrons, the second peak charge at 2.4 V involving 2 electrons, the first discharge peak at 2.3 V e the second peak at 1.9 V both involving 2 electrons.*

Figure S11 show the thickness of S60OPT used to calculate the lithium diffusion coefficient with Randles-Sevcik equation. See manuscript figure 7(f).

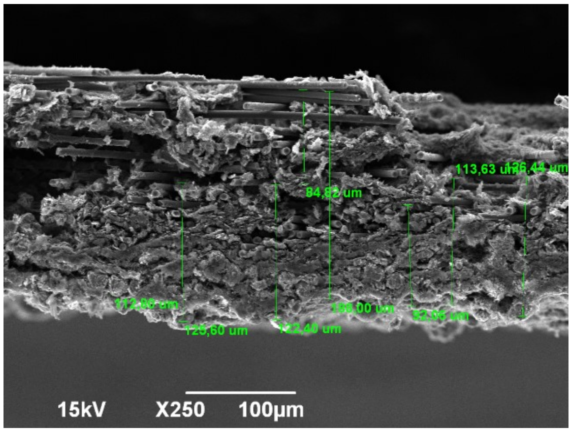


*Figure S11- SEM images of electrode thickness of S60OPT used to calculate the lithium diffusion coefficient with Randles-Sevcik equation.*
